# The Resident Medical Officers and the Nursing Staff

**Published:** 1908-04-11

**Authors:** 


### Resident Medical Officers' Department.

### THE RESIDENT MEDICAL OFFICER AND THE NURSING STAFF.

The writer has adopted the above subject as a suitable one for this department of The Hospital in the hope that its discussion may prove of use to some who may be entering upon hospital appointments.

Now it behoves all men and women holding office to appreciate duly the exact range of their own official duties and their relationship to other officers and their duties. It has been said, and perhaps unkindly, that no woman holds power without insisting to the full upon all the privileges that appertain to her position, and at times intruding upon those of others whose scope is outside the boundaries of her province. It will be well for the sisters and head nurses of large hospitals, and the matrons of smaller hospitals, and resident medical officers to realise that their duties and relations must always in the main be so regulated that the benefit of the patient, whose presence and treatment are their *raison d'être*, is the chief end in view. Such duties fall roughly under three great headings—firstly, those peculiar to the nurse; secondly, those peculiar to the resident; thirdly, those which belong partly to both officers. In large hospitals written rule and unprinted custom have laid down more or less clearly the lines of the first two sets of duties; in smaller hospitals the bounds may by mutual consent become more elastic according to the exigencies of numerical strength and circumstance. But it is in the third set that difference is likely to ensue. In large hospitals the sister works under higher officers, who in the main direct her duties and to whose authority she may appeal; the resident, too, holds rank beneath seniors, and his work is often the mere completion of another's directions: but both have multifarious duties to perform, emergencies to meet, and arrangements to make which lie wholly with themselves. Yet in these it must never be forgotten that the resident is *locum tenens* for the head physician or surgeon, and, as such, holds the main responsibility for the patients, and that no essential alteration of any detail of treatment should be undertaken without his consent. In a smaller hospital the authority of the resident is correspondingly greater, especially since the experience and capacity of the nursing staff is often less. We would then point out the necessity for each to recognise the limit of the other's duties, and to meddle nowise therein unless glaring discrepancy leaves no alternative; for each to respect the other's authority, to support it in their presence, and to uphold it in their absence; and for both on neutral ground to conspire (together) to their mutual benefit. Let the sister of long experience yield without grudging the helpful suggestions which ripe wisdom may prompt and long service justify, while carrying out with unquestioning fidelity directions for treatment whether staled by custom or staggering in their novelty. Let the resident be methodic in his visits, and duly respect ward meal-times and cleanings. It is for him to be decisive in treatment and assiduous in superintending its execution without undue interference or unseemly cavil. Let both consider pompous officiousness as the deadly sin, and yield with a good grace if untempered zeal has outstripped discretion, or if trespass upon the other's office has accompanied inexperience; let each sink the ego of desire in the altruism of their work; and, lastly, let them so labour together as to combine peace with honour.

